# Measurements of 6-thioguanine nucleotide levels with *TPMT* and *NUDT15* genotyping in patients with Crohn’s disease

**DOI:** 10.1371/journal.pone.0188925

**Published:** 2017-12-05

**Authors:** Ji Hyeon Lee, Tae Jun Kim, Eun Ran Kim, Sung Noh Hong, Dong Kyung Chang, Li-Hwa Choi, Hye In Woo, Soo-Youn Lee, Young-Ho Kim

**Affiliations:** 1 Department of Medicine, Samsung Medical Center, Sungkyunkwan University School of Medicine, Seoul, Republic of Korea; 2 Department of Laboratory Medicine, Samsung Medical Center, Sungkyunkwan University School of Medicine, Seoul, Republic of Korea; 3 Department of Laboratory Medicine, Green Cross Laboratories, Yongin, Gyeonggi, Republic of Korea; 4 Department of Laboratory Medicine, Samsung Changwon Hospital, Sungkyunkwan University School of Medicine, Changwon, Republic of Korea; University Hospital Llandough, UNITED KINGDOM

## Abstract

The association between the 6-thioguanine nucleotide (6-TGN) level and clinical remission in Crohn’s disease (CD) remains controversial. Thiopurine-induced leukopenia is a life-threatening complication of CD in Asians that was recently shown to strongly correlate with *NUDT15* genetic variants. This study aimed to determine the relationship between thiopurine metabolite levels and therapeutic response, and to investigate the association of *NUDT15*, *TPMT*, and thiopurine metabolites with leukopenia in patients with CD. We enrolled 165 adult patients with CD undergoing thiopurine treatment. Clinical evaluation and laboratory examinations were carried out every 2–3 months. We measured thiopurine metabolites levels and genotyped *NUDT15* and *TPMT*. During the median 12-month observational period, 95 (67.9%) patients exhibited clinical response and 45 (32.1%) did not respond to the treatment. The median 6-TGN level was significantly higher in responders than in non-responders (*P* < 0.001). The odds ratio of patients with a 6-TGN level ≥230 pmol/8 × 10^8^ red blood cells for showing a clinical response was 4.63 (95% CI 1.62–11.9). *NUDT15* variant types were strongly associated with developing leukopenia. Patients with *NUDT15* homozygous variant genotype developed severe early leukopenia with an average reduction of 88.2% (range, 84–94%) from the baseline white blood cell count at 4 weeks. Our findings support the role of therapeutic drug monitoring in thiopurine maintenance treatment to optimize thiopurine therapy, especially, for non-responding CD patients. Thiopurine treatment should not be recommended to patients with *NUDT15* homozygous variant genotype due to severe early leukopenia.

## Introduction

Thiopurines, such as azathioprine (AZA) and 6-mercaptopurine (6-MP), are drugs used in the induction and maintenance therapy of Crohn’s disease (CD) [1,2]. AZA and 6-MP have no intrinsic activity, so both drugs must undergo extensive metabolic transformations, resulting in 6-thioguanine nucleotides (6-TGN) and 6-methylmercaptopurine nucleotides (6-MMPN), which are considered to be the primary active metabolites of these drugs [3]. While 6-TGN and 6-MMPN are responsible for therapeutic efficacy, they exert undesirable myelosuppressive effects, and the 6-MMPN level is also associated with hepatotoxicity of 6-MP and AZA [4,5].

Thiopurine S-methyltransferase (*TPMT*) is the key enzyme in the metabolic pathway of thiopurine compounds. Previous studies have shown that lower *TPMT* activity is associated with higher 6-TGN and a higher likelihood of clinical response to thiopurine therapy, but also higher risk for leukopenia [6–9]. However, only about a quarter of inflammatory bowel disease (IBD) patients with thiopurine-induced leukopenia carry a *TPMT* mutation [10–12]. Moreover, while the frequency of *TPMT* mutations is lower in Asians (1–3%) than in Caucasians (~10%), the occurrence of thiopurine-induced leukopenia is considerably higher in Asians [13–16]. In addition to the previously known *TPMT* mutation, *NUDT15* (nucleoside diphosphate-linked moiety X-type motif 15) genetic variants have been recently identified to be strongly associated with thiopurine-induced leukopenia in Asians [17–19]. Yang et al. reported that the *NUDT15* allele encoding p.Arg139Cys was found in 89.4% of early leukopenia cases but in only 6.8% of controls, suggesting that the presence of the *NUDT15* allele had a sensitivity of 89.4% and specificity of 93.2% for early leukopenia in Koreans [17].

The aim of this study was to assess the association between thiopurine metabolite levels and therapeutic response and to evaluate the association of *NUDT15*, *TPMT*, and metabolites of thiopurines with leukopenia in patients with Crohn’s disease (CD). In addition, we evaluated factors that predict 6-TGN concentration and clinical response.

## Materials and methods

### Patients and data collection

This retrospective study included 165 patients with CD older than 20 years of age at Samsung Medical Center, South Korea, from January 2014 to March 2015. Patients received a consistent dose of AZA or 6-MP for at least 3 months prior to measurement of thiopurine metabolite levels. Patients with leukopenia or elevated liver enzyme levels before treatment with thiopurines and pregnant women were excluded. Additionally, patients with 6-thioguanine metabolite levels that had been measured within 12 h of taking AZA or 6-MP and patients taking anti-TNF drugs were excluded. We measured the patients’ levels of thiopurine metabolites, including 6-TGN and 6-MMPN, at baseline. Initially, patients received AZA at 0.5–1.0 mg/kg (6-MP 0.25–0.5 mg/kg), and this dose was increased by 0.5 mg/kg (6-MP 0.25 mg/kg) every 1–2 weeks until the target dose was reached. Clinical evaluation and laboratory examinations were performed every 2–3 months according to the clinical condition of the patient during the median 12-month observational period. Clinical and demographic data, including age, sex, weight, diagnosis, AZA or 6-MP dosage in mg/kg of body weight per day, concomitant drug treatments, and toxicity, were collected at every visit. The following laboratory data were assessed: counts of leukocytes, neutrophils, lymphocytes, and platelets; levels of alanine aminotransferase, aspartate aminotransferase, C-reactive protein (CRP); and erythrocyte sedimentation rate (ESR). In addition, blood samples were collected for determination of 6-TGN and 6-MMPN metabolite levels and for *TPMT* genotyping. This study was approved by the Institutional Review Board of Samsung Medical Center and was conducted in accordance with the Declaration of Helsinki. Written informed consent was obtained from all patients.

### Outcome definition

Primary outcomes included correlations between thiopurine metabolite levels and CD activity index (CDAI) scores and a calculated therapeutic 6-TGN cutoff level. CDAI score was calculated at every visit. Therapeutic response and non-response were determined according to these scores. Therapeutic response was defined as patients with remission, which was determined by a CDAI score <150 without a step-up therapy of anti-TNF agents during the follow-up period. Therapeutic non-response was defined as patients with active disease, which was determined by a CDAI score ≥150 with or without a step-up therapy of anti-TNF agents during the follow-up period. Additionally, we defined the therapeutic response using CRP cutoff point 0.6 mg/dl instead of CDAI score. A 6-TGN concentration of 235–450 pmol/8 × 10^8^ red blood cells (RBC) was considered the optimal therapeutic range. Poor compliance was defined as a 6-TGN level below 100 pmol/8 × 10^8^ RBC in the absence of a metabolite profile suggesting hypermethylation of thiopurines to MMPN (6-MMPN/6-TGN >11) [20]. We also evaluated predictors of 6-TGN levels and clinical response.

The criteria for toxicity were as follows: leukopenia (leukocyte count < 3 × 10^9^/l), neutropenia (neutrophil count < 1.5 × 10^9^/l), lymphopenia (lymphocyte count < 1 × 10^9^/l), thrombocytopenia (platelet count < 150 × 10^9^/l), hepatotoxicity (alanine aminotransferase or aspartate aminotransferase enzyme activity more than twice the upper limit of normal), gastrointestinal discomfort (diarrhea, nausea, or vomiting), skin rash (significant redness of the skin), and alopecia (new onset of hair loss after starting AZA or 6-MP).

### Thiopurine metabolite assays

Blood samples for determination of thiopurine metabolite levels were obtained at least 12 h after thiopurine treatment. 6-TGN and 6-MMPN RBC levels were measured on an API4000 tandem mass spectrometer (Applied Biosystems, Foster City, CA, USA) equipped with an Agilent Technologies Series 1200 HPLC system (Agilent Technologies, Santa Clara, CA, USA) [21]. Intra- and inter-assay coefficients of variation for thiopurine metabolite measurements were below 10%. The lower detection limits for quantification were 0.1 mol/l for 6-TGN and 0.5 mol/L for 6-MMPN.

### *TPMT* and *NUDT15* genotyping

After peripheral blood samples were obtained, genomic DNA was extracted from the peripheral blood leukocytes of patients using a Wizard Genomic DNA Purification Kit (Promega, Madison, WI, USA) according to the manufacturer’s instructions. We genotyped four SNPs in the *TPMT* gene (rs1142345 for *3, rs75543815 for *6, rs144041067 for *16, rs115106670 for *32) using the MassARRAY Analyzer Compact System and MassARRAY Typer software ver. 4.0.2 (Sequenom, Inc., San Diego, CA, USA).[22,23] Exons 1 and 3 of *NUDT15* and their intron–exon boundaries were amplified by PCR using primers designed in-house. For exon 3, PCR was conducted on a GeneAmp PCR System 9700 (Applied Biosystems) with 35 cycles of 94°C for 20 s, 60°C for 30 s, and 72°C for 40 s. For exon 1, an Eppendorf pro S (Eppendorf) was used, with 50 cycles of 95°C for 20 s, 62°C for 30 s, and 72°C for 40 s. Five microliters of the amplicon of both exons was treated with 10 U exonuclease I (USB Corp., Cleveland, Ohio, USA) at 37°C for 15 min and incubated at 80°C for 15 min to inactivate the enzyme. Cycle sequencing of exons 1 and 3 of *NUDT15* (NCBI reference sequence NM_018283.3) was performed using Big Dye Terminator Cycle Sequencing Ready Reaction kits (Applied Biosystems) on an ABI 3130xl genetic Analyzer (Applied Biosystems) [24].

### Statistical analysis

Continuous variables are reported as the mean ± standard deviation (SD), while categorical variables are presented as percentages. Differences between continuous variables were compared using Student’s *t*-test or Mann-Whitney *U*-test, while differences between categorical variables were analyzed using Chi-squared or Fisher’s exact test. To evaluate the precise association between 6-TGN levels and clinical remission, we conducted univariable and multivariable analyses after excluding non-compliant patients. Univariable and multivariable regression analyses were performed to evaluate factors that predict 6-TGN levels. The variables analyzed included age, sex, BMI, 5-aminosalicylic acid (5-ASA) use, steroid use, anti-TNF use, presence of leukopenia, thiopurine dosage, duration of thiopurine treatment, and *TPMT* genotype. The predictive value of the 6-TGN level for clinical response and adverse event was evaluated using receiver operating characteristic (ROC) curves. The area under the curve (AUC), Youden’s index, sensitivity, and specificity were calculated. Youden’s index is the difference between the true positive rate (sensitivity, %) and the false positive rate (1 –specificity, %). Finding the point on the ROC curve that maximizes Youden’s index provides an optimal cutoff value [25]. Multivariable logistic regression analyses were performed to evaluate factors that predict clinical response and leukopenia. *P* < 0.05 was considered statistically significant. Statistical analyses were performed using SAS version 9.4 (SAS Institute, Cary, NC, USA).

## Results

### Patient characteristics

One hundred forty patients with CD, aged 20 years or older, were included in the analysis. The mean (SD) age of the patients was 33.6 (8.8) years. Patients were treated with thiopurine (mean dose, AZA: 1.2 mg/kg/day, 6-MP: 0.6 mg/kg/day) for a period ranging from 7 to 96 months (median = 34 months). During the median 12 months (ranging from 5 to 22 months) of the observational period, 95 (67.9%) patients showed clinical remission based on CDAI score, and 98 (70%) patients showed clinical remission based on CRP level. Patient characteristics are shown in Table 1.

**Table 1:** Characteristics of patients.

	Total(n = 140)	Responder (n = 95)	Non-responder (n = 45)	*P* value
**Age, year(mean ± SD)**	33.6 ± 8.8	33.1 ± 8.5	34.5 ± 9.3	0.44
**Sex, n (%)**				0.09
**Male**	114 (81.4)	81 (85.3)	33 (73.3)	
**Female**	26 (18.6)	14 (14.7)	12 (26.7)	
**BMI (m^2^/Kg)**	21.5 ± 3.8	22.3 ± 3.3	20.0 ± 4.3	0.001
**CDAI**	111 ± 89	61 ± 39	219 ± 41	<0.001
**Location of CD, n (%)**				0.34
**Ileal**	11 (7.9)	8 (8.4)	3 (6.7)	
**Ileocolonic**	48 (34.3)	36 (37.9)	12 (26.7)	
**Colonic**	81 (57.9)	51 (53.7)	30 (66.7)	
**Type of thiopurine, n (%)**				0.27
**Azathioprine**	132 (94.3)	91 (95.8)	41 (91.1)	
**6-mercaptopurine**	8 (5.7)	4 (4.2)	4 (8.9)	
**Thiopurine start dose (mg.kg**^**-1**^**.d**^**-1**^**)**				
**Azathioprine**	0.8 ± 0.5	0.8 ± 0.5	0.8 ± 0.4	0.26
**6-mercaptopurine**	0.4 ± 0.2	0.4 ± 0.2	0.4 ± 0.1	0.81
**Thiopurine maintenance dose** **(mg.kg**^**-1**^**.d**^**-1**^**)**				
**Azathioprine**	1.2 ± 0.5	1.3 ± 0.5	1.0 ± 0.5	0.01
**6-mercaptopurine**	0.6 ± 0.3	0.6 ± 0.3	0.6 ± 0.2	0.92
**Duration of thiopurine, month**	35.7 ± 19.6	35.2 ± 19.2	36.9 ± 19.7	0.32
**Co-medication, n (%)**				
**5-ASA**	63 (45.0)	58 (61.1)	5 (11.1)	<0.001
**Steroid**	2 (1.4)	1 (1.1)	1 (2.2)	0.54
***TPMP* genotype**				0.18
*1/*1	135 (96.4)	90 (94.7)	45 (100)	
*1/*3	5 (3.6)	5 (5.3)	0 (0)	
**Toxicity**				
**Leukopenia**	38 (27.1)	29 (30.5)	9 (20.0)	<0.001
**Neutropenia**	24 (17.1)	18 (18.9)	6 (13.3)	<0.001
**Lymphopenia**	53 (37.9)	41 (43.1)	12 (26.7)	<0.001
**Hepatitis**	1 (1.3)	1 (2.1)	0 (0)	1.000
**GI intolerance**	13 (9.3)	8 (8.4)	5 (11.1)	0.76
**Skin rash**	2 (1.4)	1 (1.1)	1 (2.2)	0.54
**Alopecia**	2 (1.4)	1 (1.1)	1 (2.2)	0.54

Values are expressed as means ± standard deviation or percentages.

BMI, body mass index; CDAI, Crohn’s disease activity index; CD, Crohn’s disease; 5-ASA, 5-aminosalicylic acid; *TPMT*, thiopurine S-methyl8transferase; GI, gastrointestinal.

During the study period, several adverse drug reactions were observed. The most common side effect was lymphopenia (37.9%). Leukopenia, neutropenia, and lymphopenia were more common in responders than in non-responders. Incidence rates of other toxic events did not differ between both groups. There were five (5.3%) responders with a variant *TPMT* genotype (*1/*3), while there were no non-responders with variant genotypes.

### Thiopurine metabolite levels

Based on CDAI score, 47 (49.5%) patients in the responder group and 8 (17.8%) patients in the non-responder group had 6-TGN concentrations within the optimal range (235–450 pmol/8 × 10^8^ RBC) (Table 2). Based on CRP level, 51 (52%) patients in the responder group and 4 (9.8%) patients in the non-responder group had optimal 6-TGN concentrations. The median 6-TGN concentration among responders was significantly higher than that of non-responders. The median 6-MMPN level of responders was also significantly higher than that of non-responders, particularly based on the CRP criteria. The non-responder group had a higher proportion of non-compliant patients (6-TGN levels < 100 pmol/8×10^8^ RBC) than the responder group. Patients that developed leukopenia had higher levels of 6-TGN than those without leukopenia. The median 6-TGN levels of patients with a variant *TPMT* genotype were significantly higher than those of patients with wild-type *TPMT*. In contrast, there was no difference in the median 6-TGN level between patients harboring variant *NUDT15* and those carrying wild-type *NUDT15*. The 5-ASA users exhibited higher levels of 6-TGN and lower ratios of 6-MMPN/6-TGN than 5-ASA non-users (*P* < 0.001).

**Table 2:** Thiopurine metabolites levels in different subgroups.

	Responder	Non-responder	*P* value
***By CDAI (150)***	N = 95	N = 45	
**Number of 6-TGN 235–450 level (%)**	47 (49.5)	8 (17.8)	< 0.001
**6-TGN (pmol/8x10**^**8**^ **RBC)**	299.9 (226–393)	176.8 (124–220)	< 0.001
**6-MMPN (pmol/8×10**^**8**^ **RBC)**	428.1 (236–792)	410 (206–780)	0.12
**Number of poor-compliant patients (%)**	2 (5.3)	10 (22.2)	< 0.001
***By CRP (0*.*6 mg/dl)***	N = 98	N = 41	
**Number of 6-TGN 235–450 level (%)**	51 (52.0)	4 (9.8)	< 0.001
**6-TGN (pmol/8×10**^**8**^ **RBC)**	314.9 (239–401)	174 (127–206)	< 0.001
**6-MMPN (pmol/8×10**^**8**^ **RBC)**	421.7 (220–837)	355.6 (169–584)	0.027
**Number of poor-compliant patients (%)**	4 (4.1)	8 (19.5)	< 0.001
	**Leukopenia (n = 26)**	**Normal (n = 114)**	
**6-TGN (pmol/8×10**^**8**^ **RBC)**	330.1 (210–549)	230.5 (151–325)	0.002
**6-MMPN (pmol/8×10**^**8**^ **RBC)**	410 (220–742)	355.6 (186–708)	0.625
	**Variant type *TPMT* (n = 5)**	**Wild type *TPMT* (n = 135)**	
**6-TGN (pmol/8×10**^**8**^ **RBC)**	302 (232–399)	253.1 (174–379)	0.01
**6-MMPN (pmol/8×10**^**8**^ **RBC)**	475.3 (242–625)	359.3 (186–668)	0.55
	**Variant type *NUDT15* (n = 22)**	**Wild type *NUDT15* (n = 97)**	
**6-TGN (pmol/8×10**^**8**^ **RBC)**	280.8 (194–376)	253.1 (162–350)	0.622
**6-MMPN (pmol/8×10**^**8**^ **RBC)**	369.0 (199–779)	359.2 (206–757)	0.692
	**5-ASA users (n = 63)**	**5-ASA non-users (n = 77)**	
**6-TGN (pmol/8×10**^**8**^ **RBC)**	332.9 (252–536)	193.8 (112–262)	<0.001
**6-MMPN (pmol/8×10**^**8**^ **RBC)**	359.3 (211–745)	407 (202–792)	0.83

Values are expressed as median (interquartile range) or percentages.

CDAI, Crohn’s disease activity index; 6-TGN, 6-thioguanine nucleotides; 6-MMPN, 6-methylmercaptopurine nucleotides; CRP, C-reactive protein; *TPMT*, thiopurine S-methyltransferase; 5-ASA, 5-aminosalicylic acid.

### Predictors of 6-TGN levels

Univariable analysis showed that concurrent use of 5-ASA, presence of leukopenia, azathioprine/mercaptopurine weight-based dosage, and *TPMT* variant genotype were significantly associated with 6-TGN levels (S1 Table). In the multivariable analysis, 5-ASA use, AZA dose, and variant *TPMT* genotype were significant predictors of high 6-TGN levels (*P* < 0.001). The relationship between thiopurine dosage and 6-TGN level was significant (β coefficient, 0.407; 95% CI, 0.281–0.523). Age, sex, BMI, concurrent use of steroids, and duration of thiopurine treatment were not associated with 6-TGN levels.

### 6-TGN levels and clinical response

Multivariable logistic regression analyses revealed that BMI levels, concurrent use of 5-ASA, and 6-TGN levels were significantly associated with therapeutic response (Table 3). The odds ratio (OR) for having a clinical response with 6-TGN levels ≥230 pmol/8 × 10^8^ RBC was 4.63 (95% CI, 1.62–11.9) when compared with lower 6-TGN levels (<230 pmol/8 × 10^8^ RBC). We determined the ROC curve of 6-TGN levels for clinical response. The AUC of 6-TGN levels was 0.771 for therapeutic response by CDAI and 0.801 for therapeutic response by CRP (*P* < 0.001). The highest Youden’s index was observed when the cutoff value was 230.2. The sensitivity and specificity of the 230.2 cutoff were 71.6% and 77.8%, respectively. The clinical response rate was 68/77 (88.3%) based on a threshold 6-TGN level of 230.2 (pmol/8 × 10^8^ RBC). In addition, leukopenia and lymphopenia were noted in 44.4% and 61.1% of patients with toxic levels of 6-TGN >450 pmol/8 × 10^8^ RBC, respectively. In patients with non-toxic 6-TGN levels, rates of leukopenia and lymphopenia were 24.6% and 34.4%, respectively. The AUC of 6-TGN levels was 0.723 for leukopenia (*P* < 0.001). The highest Youden’s index was observed when the cutoff value was 330. The sensitivity and specificity of the 330 cutoff were 59.1% and 77.8%, respectively.

**Table 3:** Association 6-TGN levels and clinical response (multivariable logistic regression analyses).

	Multivariable analysis	
	OR (95% CI)	*P* value
**Age**	0.97 (0.92–1.03)	0.27
**Female sex**	1.33 (0.39–4.55)	0.65
**BMI**	1.24 (1.05–1.46)	0.01
**5-ASA co-medication**	1.64 (1.21–2.19)	0.006
**Steroid co-medication**	0.21 (0.01–5.30)	0.35
**6-TGN levels**		
**6-TGN (continuous)**	1.02 (1.01–1.03)	0.007
**6-TGN ≥ 230 (binary)**	4.63 (1.62–11.9)	0.004
**Duration of thiopurine treatment**	1.01 (0.99–1.02)	0.79
***TPMT* variant genotype**	1.33 (0.12–5.58)	0.87

OR, odds ratio; CI, confidence interval; BMI, body mass index; 5-ASA, 5-aminosalicylic acid; TNF, tumor necrosis factor; 6-TGN, 6-thioguanine nucleotide; TPMT, thiopurine S-methyltransferase.

**Fig 1:**
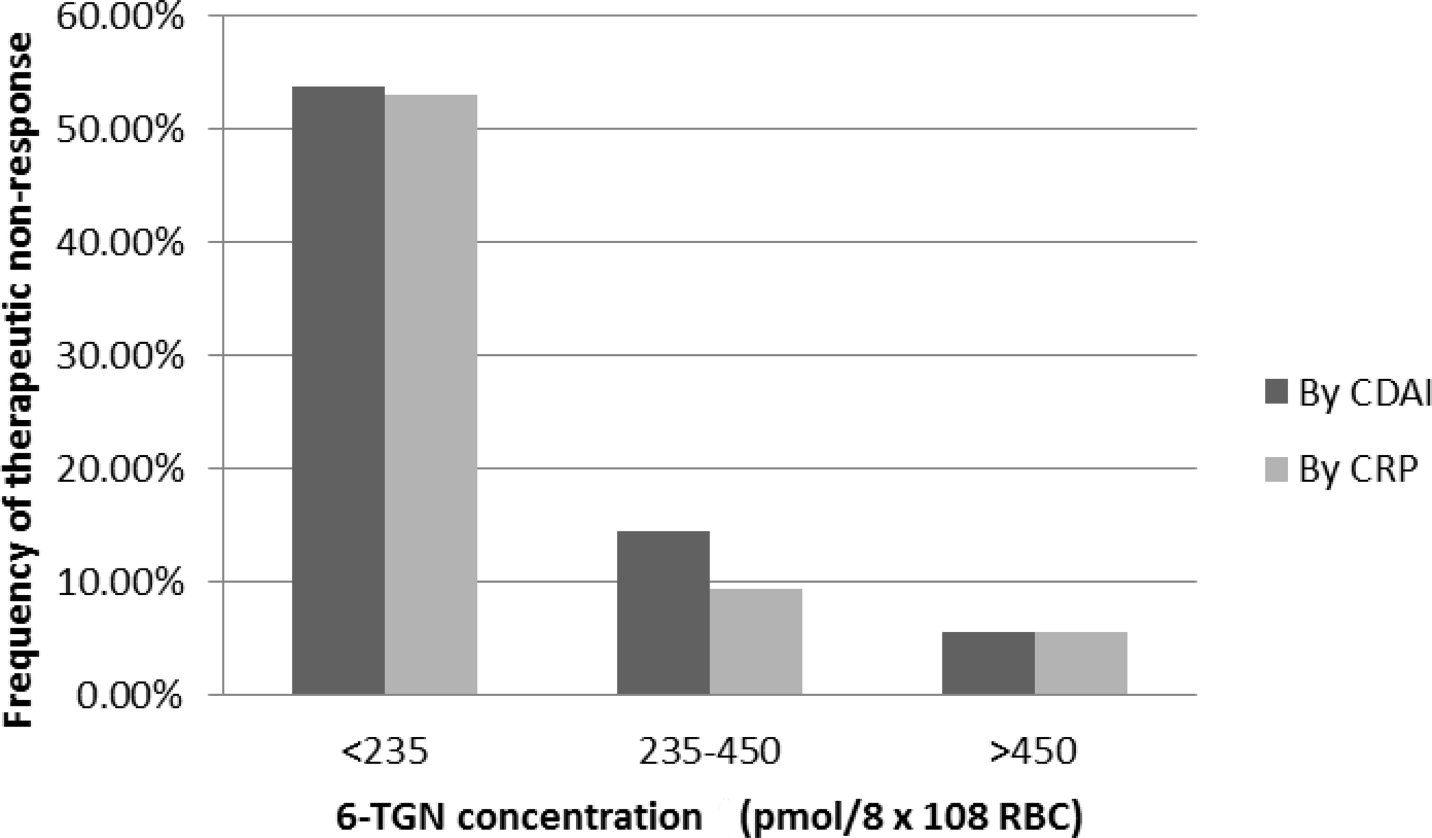
The frequency of patients with therapeutic non-response according to 6-TGN concentrations.

When patients were divided into three groups according to 6-TGN levels (<235, 235–450, and >450 pmol/8 × 10^8^ RBC), the frequency of therapeutic non-response was significantly higher in patients with 6-TGN levels below 235 pmol/8 × 10^8^ RBC (Fig 1).

### *NUDT15* and *TPMT* genotypes and leukopenia

**Fig 2:**
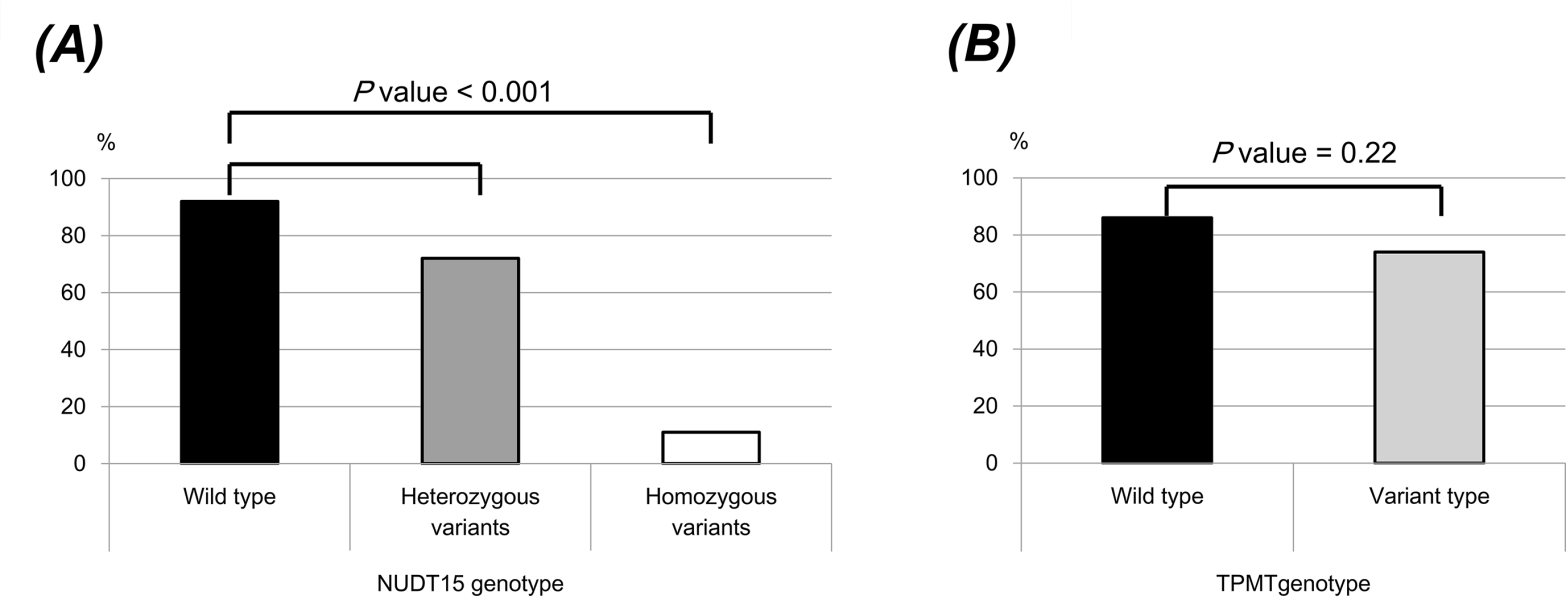
WBC count as a percentage of baseline at 4 weeks after thiopurines treatment; (A) NUDT15 genotype, (B) TPMT genotype. CDAI, Crohn’s disease activity index; CRP, C-reactive protein; 6-TGN, 6-thioguanine nucleotides.

Out of 165 patients, 34 (20.6%) had *NUDT15* variant alleles. *NUDT15* heterozygous variants were found in 28 patients (17%) and *NUDT15* homozygous variants were found in 6 patients (3.6%). The allele frequencies of wild type *NUDT15* was 87.2% and the frequency of variant alleles was 12.8%. Table 4 shows the risk of leukopenia according to *NUDT15* and *TPMT* genotypes. *NUDT15* variant types were strongly associated with developing leukopenia [10.9% wild-type as reference; 48.5% heterozygous variant genotype; 100% homozygous variant genotype, OR 3.44 (95% CI, 1.21–9.78)]. However, *TPMT* genotype was not associated with developing leukopenia (OR 0.58, 95% CI 0.07–4.96). Six patients with *NUDT15* homozygous variant genotype developed severe early leukopenia with average reduction of 88.2% (range, 84–94%) from the baseline WBC count at 4 weeks (Fig 2 and S2 Table). Among them, 4 patients developed severe neutropenia with sepsis and 1 patient developed alopecia totalis. They stopped thiopurine and received antibiotic treatment. All 4 patients recovered.

**Table 4:** The risk of leukopenia according to *NUDT15* and *TPMT* genotype.

	Frequency of early leukopenia (0–8 weeks)	Frequency of leukopenia (0–48 weeks)		OR (95% CI)	
				Early leukopenia (0–8 weeks)	Leukopenia (0–48 weeks)
***NUDT15***					
**Wild**	0.8%	10.9%		1.00 (reference)	1.00 (reference)
**Heterozygous variant**	24.2%	48.5%		26 (2.81–241.10)	3.44 (1.21–9.78)
**Homozygous variant**	100%	100%			
***TPMT***					
**Wild**	8.7%	21.1%		1.00 (reference)	1.00 (reference)
**Variant**	14.3%	14.3%		1.89 (0.21–9.98)	0.58 (0.07–4.96)

*TPMT*, thiopurine S-methyltransferase; OR, odds ratio; CI, confidence intervals.

## Discussion

In this study of CD patients on thiopurine maintenance therapy, we showed that patients with higher levels of 6-TGN have a significantly higher chance of being in remission. We also found that the optimal cutoff value of 6-TGN was 230.2 pmol/8 × 10^8^ RBC. Patients with 6-TGN levels ≥230 pmol/8 × 10^8^ RBC had a 4.6 times greater chance of clinical response than those with 6-TGN levels <230 pmol/8 × 10^8^ RBC. In addition, *NUDT15* variant types were strongly associated with developing leukopenia.

The association between 6-TGN and clinical remission remains a matter of debate in IBD. Several studies have suggested that 6-TGN metabolite levels cannot be used to predict remission. Gonzalez et al. evaluated 113 patients in a prospective multicenter study and reported that neither *TPMT* activity nor 6-TGN concentration predicted treatment outcome [26]. Gilissen et al. found that median 6-TGN levels did not significantly differ between exacerbation and remission groups [3]. In contrast to these findings, several other studies have reported that the monitoring of 6-TGN concentrations may be helpful for managing AZA or 6-MP therapy in IBD patients, as it may identify the optimal AZA/6-MP dose to maximize efficacy while minimizing the risk of toxicity [4,21,27]. A recent meta-analysis demonstrated that patients with 6-TGN levels above a predefined cutoff were three times more likely to experience remission, with a pooled OR of 3.15 (95% CI, 2.41–4.11) [28]. In our study, we found that the optimal 6-TGN level was 230 pmol/8 × 10^8^ RBC; patients with 6-TGN levels above this cutoff were 4.6 times more likely to exhibit clinical responses.

*TPMT* genotyping prior to thiopurine treatment in patients with IBD has been shown to substantially reduce the risk of adverse effects without compromising therapeutic efficacy [29–31]. In Asian populations, including Koreans, the frequencies of *TPMT* mutations are lower than those reported from Western countries [13–16]. Among a total of 900 Korean patients genotyped for *TPMT*, 30 patients (3.3%) had known TPMT variant alleles [22]. In our study, 5 out of 140 patients were genotyped as *1/*3 (3.6%), and we also found a correlation between *TPMT* genotype and 6-TGN metabolite levels. A recent genome-wide association study identified a missense variant in the *NUDT15* gene that is strongly associated with thiopurine-related myelosuppression in patients with IBD [17]. The *NUDT15* genotype frequencies in our study (wild type, 79.4%; variant type, 20.6%) were similar to those recently reported in studies on Koreans and Japanese, and *NUDT15* variant genotype is much more common than *TPMT* mutation [17,32]. Several studies have reported an association between *NUDT15* and thiopurine-induced leukopenia [33–35]. Our data also showed that leukopenia was more prevalent in patients with variant *NUDT15* than in patients with wild-type *NUDT15*. In addition, 6 patients who could not tolerate thiopurine therapy because of severe neutropenic events harbored the homozygous *NUDT15* variant genotype. These findings are consistent with recent reports and imply pretreatment determination of the *NUDT15* genotype is necessary to identify IBD patients susceptible to thiopurine-induced leukopenia [17–19]. We recommend that treatment with thiopurine should be avoided in patients with *NUDT15* homozygous variants, and in cases with heterozygous variants, slow dosage increase rather than the usual schedule, with careful monitoring of adverse effects, is highly desirable.

Thiopurine-induced leukopenia has been primarily explained by *TPMT* variants that induce the intracellular 6-TGN accumulations [36]. Recent studies found *NUDT15* mutation as another critical determinant of thiopurine-induced leukopenia. *NUDT15* inactivates thiopurine metabolites by converting thio-guanosine triphosphate (TGTP) to TGMP, thus preventing their incorporation into DNA and negatively affecting desired cytotoxic effects of thiopurines [37]. Theoretically, patients with defective *NUDT15* alleles can have excessive levels of thiopurine metabolites and their toxicity. In our study, *NUDT15* variants-related leukopenia was not accompanied by an elevation of 6-TGN levels, it may tend to affect the level of TGTP and TdGTP rather than total 6-TGN [38]. Two recent studies in Asia also reported that there was no significant difference in 6-TGN level among the *NUDT15* genotypes [18,35]. Therefore, patients with the *NUDT15* variants may call for a new therapeutic monitoring method to detect the active metabolites TGTP rather than 6-TGN to explain the mechanism of *NUDT15*-related leukopenia.

The mean AZA weight-based dosage in this study was 1.2 ± 0.5 mg∙kg^-1^∙d^-1^, which is much lower than the recommended dose according to most guidelines. A daily AZA dose of 2–3 mg/kg for individuals with wild-type *TPMT* is recommended by the Clinical Pharmacogenetics Implementation Consortium guidelines [39], and 1.5 to 2.5 mg∙kg^-1^∙d^-1^ is recommended by the European Crohn’s and Colitis Organization [40]. Several studies have reported that lower doses of thiopurines exert sufficient clinical efficacy and result in therapeutic concentrations of 6-TGN in Japanese IBD patients [41–43]. The reason for the lower dose of AZA in Asian population can be explained by the frequency of the *NUDT15* variants allele. The frequency of the wild-type allele of *NUDT15* was higher in African and European populations (>99.0%) than in Asian populations (82.8–93.0%) [24]. The frequency (12.8%) of *NUDT15* variant alleles in our study was comparable with that reported for Asian populations in previous studies. Therefore, our findings along with several previous studies suggest that appropriate thiopurine doses for Asian populations may be lower than those recommended by current guidelines [44].

5-ASA is generally used in the induction and maintenance of remission of ulcerative colitis. The studies evaluating the effect of 5-ASA as induction and maintenance treatment in Crohn’s disease are controversial and the Cochrane database stated that 5-ASA was not effective in the treatment of Crohn’s disease [45]. In this study, 5-ASA co-medication along with azathioprine was associated with improved clinical response. The efficacy of 5-ASA in combination with azathioprine may be due to the additive effect of 5-ASA. Previous studies revealed that thiopurine therapy in combination with 5-ASA led to increased 6-TGN levels in 82% to 100% of study patients, which suggests increased therapeutic efficacy [46,47]. Another study provided the evidence that 5-ASA are potent noncompetitive inhibitors of *TPMT* enzyme activity by strong affinity for *TPMT*, which results in increased 6-TGN accumulation and consequently a boost to the immunosuppressive effect [48].

This study had some limitations. First, it was a single-center, retrospective study and thus, carries certain limitations compared with a prospective design, such as lacking strictly controlled medical visit schedules and missing clinical or laboratory variables. Therefore, there is a need for additional prospective studies that follow patients on thiopurine treatment from the start of therapy and assess the efficacy and toxicity at several time points through monitoring of thiopurine metabolite levels. A previous prospective study on individualized dosing based on 6-TGN concentrations showed negative results; however, the statistical power was low owing to inadequate enrollment and early withdrawals [49]. A prospective study with adequate sample size and follow-up period to compare the clinical efficacy of dose adjustments based on thiopurine metabolite levels with standard dose adjustments based on white blood cell counts would also be informative.

In conclusion, our results show that patients with higher 6-TGN levels exhibit a significantly higher chance of being in remission. The optimal threshold level of 6-TGN for therapeutic response was 230 pmol/8\10^8^ RBC. The clinical response rate was 88.3% among patients in our study who met this criterion. Combination therapy with 5-ASA was associated with higher 6-TGN levels and subsequent clinical responses. Patients with *NUDT15* variant genotypes were more likely to develop leukopenia. Therefore, these results support the role of therapeutic drug monitoring in thiopurine maintenance treatment to optimize thiopurine therapy especially for non-responding CD patients, and predetermination of *NUDT15* genotype is helpful minimize adverse events.

## Supporting information

S1 Table Predictors of 6-TGN level. (DOCX)

S2 Table Clinical and genetic characteristics of the patients who experienced severe leukopenia during thiopurine treatment. (DOCX)
