# Supplementary material for: Measurements of 6-thioguanine nucleotide levels with TPMT and NUDT15 genotyping in patients with Crohn’s disease
Source: PLoS One. 2017 Dec 5;12(12):e0188925. doi: 10.1371/journal.pone.0188925 (PMC5716599; doi:10.1371/journal.pone.0188925)
Supplement: S1 Table — (DOCX) [file pone.0188925.s001.docx]

**S1 Table. Predictors of 6-TGN level.**

|  | **Univariable Analysis** | |  | **Multivariable analysis** | |
| --- | --- | --- | --- | --- | --- |
|  | **β (95% CI)** | ***P* value** |  | **β (95% CI)** | ***P* value** |
| **Age** | -0.084 (-0.172-0.122) | 0.524 |  |  |  |
| **Female sex** | -0.126 (-0.281-0.021) | 0.137 |  |  |  |
| **BMI** | 0.06 (-0.083-0.169) | 0.502 |  |  |  |
| **5-ASA co-medication** | 0.458 (0.325-0.591) | <0.001 |  | 0.421 (0.306-0.531) | <0.001 |
| **Steroid co-medication** | -0.003 (-0.032-0.026) | 0.974 |  |  |  |
| **Azathioprine dose (mg.kg^-1^.d^-1^)** | 0.342 (0.202-0.479) | <0.001 |  | 0.412 (0.281-0.528) | <0.001 |
| **Mercaptopurine (mg.kg^-1^.d^-1^).** | 0.163 (0.011-0.362) | 0.04 |  |  |  |
| **Duration of thiopurine treatment** | 0.005 (-0.124-0.143) | 0.952 |  |  |  |
| ***TPMT* variant genotype** | 0.203 (0.061-0.348) | 0.02 |  | 0.352 (0.130-0.577) | <0.001 |

6-TGN, 6-thioguanine nucleotides; β, the estimated coefficient; CI, confidence interval; BMI, body mass index; 5-ASA, 5-aminosalicylic acid; TNF, tumor necrosis factor; *TPMT*, thiopurine S-methyltransferase.
