# Supplementary material for: Measurements of 6-thioguanine nucleotide levels with TPMT and NUDT15 genotyping in patients with Crohn’s disease
Source: PLoS One. 2017 Dec 5;12(12):e0188925. doi: 10.1371/journal.pone.0188925 (PMC5716599; doi:10.1371/journal.pone.0188925)
Supplement: S2 Table — (DOCX) [file pone.0188925.s002.docx]

| **Case** | **Sex** | **Age** | **Type of**  **thiopurine** | **Dose of**  **thiopurine**  **(mg/kg)** | **WBC (ANC) count at baseline** | **WBC (ANC) count at 4 weeks** | **Adverse events** | ***TPMT* gene** | **Variant type *NUDT15*** |
| --- | --- | --- | --- | --- | --- | --- | --- | --- | --- |
| 1 | F | 47 | AZA | 1.0 mg/kg | 7410 (4602) | 980 (710) |  | *1/*1 | Homozygous variant |
| 2 | F | 29 | AZA | 1.5 mg/kg | 6600 (4884) | 1020 (130) | Sepsis | *1/*3 | Homozygous variant |
| 3 | F | 20 | AZA | 1.0 mg/kg | 7940 (5272) | 610 (30) | Sepsis | *1/*1 | Homozygous variant |
| 4 | F | 19 | AZA | 0.5 mg/kg | 7230 (3868) | 450 (19) | Sepsis, alopecia totalis | *1/*1 | Homozygous variant |
| 5 | M | 21 | AZA | 1.7 mg/kg | 5790 (3620) | 930 (240) | . | *1/*1 | Homozygous variant |
| 6 | M | 41 | AZA | 1.8 mg/kg | 10030 (7050) | 1310 (140) | Sepsis | *1/*1 | Homozygous variant |

**S2 Table. Clinical and genetic characteristics of the patients who experienced severe leukopenia during thiopurine treatment.**

WBC, white blood cell; ANC, absolute neutrophil count; *TPMT*, thiopurine S-methyltransferase; AZA, azathioprine.
